# Nineteeth Annual Meeting of the Mississippi Valley Association of Dental Surgeons

**Published:** 1863-03

**Authors:** 


### Proceedings of Societies.

---

### NINETEENTH ANNUAL MEETING OF THE MISSISSIPPI VALLEY ASSOCIATION OF DENTAL SURGEONS.

The Association met according to adjournment in the first lecture room of the Ohio College of Dental Surgery, February 18, 1863, at 9 o'clock A. M. The president, H. M'Cullum, in the chair.

The Secretary being absent, E. Collins was appointed Secretary *pro tem.*

The minutes of the last annual meeting were called for and read.

Upon recommendation, Dr. W. H. Shadoan, of Rockport, Indiana, was elected a member of the association.

The following subjects for discussion were then presented.

Causes of decay and their prevention. Filling teeth. Materials employed and mode of using. Mechanical Dentistry.

On motion, the above subjects were accepted.

Moved that the society adjourn to meet at 1½ o'clock P. M.

### FIRST DAY, AFTERNOON.

Met, according to adjournment, at 1½ o'clock P. M. President in the chair.

The treasurer, Dr. Taft, presented his annual report, whereupon a committee, composed of Drs. Ulrey and Foote, were appointed to audit the accounts of the same.

Treasurer's report:

| | | | |
|---|---|---|---|
| Feb. 22, 1861. | | Received of Predecessor | $62 68 |
| " | " | Received Annual Dues for 1861 | 8 00 |
| " | " | Received from Initiation Fees | 3 00 |
| | | | $73 68 |

| | | |
|---|---|---|
| Feb. 22, 1861. | By cash paid Janitor | $ 8 00 |
| " " | Paid Recording Secretary | 10 00 |
| " " | Paid on Microscope as per order | 20 00 |
| " " | Paid for Microscopical preparations | 30 00 |
| | | $68 00 |
| | Balance on hand | $5 68 |

The reading of essays was then called for when Dr. G. F. Foote read an ably written essay, presenting, as one of the causes of decay, respiration through the mouth.

The society voted their thanks to Dr. Foote for his essay and requested of him a copy for publication.

The subject of the above essay elicited interesting and lengthy discussion, in which all the members present freely participated. Other causes of decay of the teeth were then duly considered, when discussions were opened upon Dental Hygiene.

Adjourned to meet at 9 o'clock A. M. to-morrow.

SECOND DAY—FORENOON.

Met according to adjournment. President in the chair.

The committee appointed to audit the Treasurer's account reported the same correct.

Moved, that the dues of 1862 be omitted.

Moved, that Dr. Duncan be received into membership with the association.

The society then proceeded to the election of officers, which resulted as follows:

*President*—E. Collins.

*Vice-President*—H. R. Smith.

*Recording Secretary*—H. A. Smith.

*Corresponding Secretary*—G. F. Foote.

*Treasurer*—J. Taft.

*Committee on Essays*—Drs. Foote, Taft and H. R. Smith.

*Executive Com.*—Drs. Duncan, Wheeler and M'Cullum.

*Committee on Microscope.*—Drs. Taft, H. R. Smith, G. F. Foote.

*Delegates to Dental Association.*—Drs. Foote, Collins, Ulery, M'Cullum and Duncan.

After election, President elect took the chair. Dr. H. A. Smith, secretary.

Dr. Collins both approved and disapproved of gritty substances, either in the form of paste or powder, homogeneous or heterogeneous in their composition. It mattered not if they possessed the property of removing sordes and calcareous deposits from the teeth, and of imparting to their surface a fine polish, which, having been accomplished, he then disapproved of their continued use, for the reason that they are only an evil—when used as above—rendered necessary by the presence of another evil; as any agent that possesses the property of producing attrition sufficient to remove the aforementioned substances, must, where constantly employed, result injuriously to the teeth. The medication of the above class of dentrifices he deemed unnecessary; for if the gums are in an abnormal condition, they can be more successfully treated with medicinals uncombined with such substances as do not possess remediate properties, whose sole effect is to cause additional irritation, and thus counteract, in a great measure, the good effects of the former. He approved of suitable soap dentrifices for constant use, as they serve the two fold purpose of decomposing and removing oily matters from the teeth, and of neutralizing acids.

The subjects which elicited discussion the previous day were again taken up and dealt with in the usual spirit. Dental Hygiene receiving the greater share of attention.

SECOND DAY—AFTERNOON.

Met, according to adjournment. President in the chair.

Subject of Dental Hygiene was continued at some length, and with unabated interest.

The remaining subjects were then taken up and dispensed with. Dr. Collins concluding the discussion by giving his manner of executing rubber work in combination with gold plate.

Being no further business before the meeting, it was moved to adjourn to meet again at the usual time and place next year.

